# Safety and Efficacy of a Typhoid Conjugate Vaccine in Malawian Children

**DOI:** 10.1056/NEJMoa2035916

**Published:** 2021-09-16

**Authors:** Priyanka D. Patel, Pratiksha Patel, Yuanyuan Liang, James E. Meiring, Theresa Misiri, Felistas Mwakiseghile, J. Kathleen Tracy, Clemens Masesa, Harrison Msuku, David Banda, Maurice Mbewe, Marc Henrion, Fiyinfolu Adetunji, Kenneth Simiyu, Elizabeth Rotrosen, Megan Birkhold, Nginache Nampota, Osward M. Nyirenda, Karen Kotloff, Markus Gmeiner, Queen Dube, Gift Kawalazira, Matthew B. Laurens, Robert S. Heyderman, Melita A. Gordon, Kathleen M. Neuzil

**Affiliations:** 1 Malawi-Liverpool-Wellcome Programme, Blantyre, Malawi;; 2 Center for Vaccine Development and Global Health, University of Maryland School of Medicine, Baltimore, Maryland, USA;; 3 Oxford Vaccine Group, Department of Paediatrics, Oxford University, United Kingdom;; 4 Liverpool School of Tropical Medicine, Liverpool, UK;; 5 Blantyre Malaria Project, University of Malawi College of Medicine, Blantyre, Malawi;; 6 Department of Paediatrics, Queen Elizabeth Central Hospital, Blantyre, Malawi.; 7 District Health Office, Blantyre District Council, Blantyre, Malawi;; 8 Division of Infection and Immunity, University College London;; 9 Institute of Infection, Veterinary and Ecological Sciences, University of Liverpool, United Kingdom;; 10 College of Medicine, University of Malawi, Malawi.

## Abstract

**Background:**

Typhoid fever caused by multidrug-resistant H58 *Salmonella* Typhi is an increasing public-health threat in sub-Saharan Africa. We present phase 3 efficacy data from an African trial of a Vi-polysaccharide typhoid conjugate vaccine (Vi-TCV).

**Methods:**

Children aged 9 months to 12 years in Blantyre, Malawi were randomized (1:1) in a double-blind trial to receive Vi-TCV (single dose) or group-A meningococcal control vaccine (MenA).The primary outcome was blood culture-confirmed typhoid fever. We present the primary vaccine efficacy (VE) and safety outcomes after 18–24 months of follow-up.

**Results:**

This intention-to-treat (ITT) analysis included 28,130 children, comprising 14,069 children who received Vi-TCV and 14,061 children who received MenA. Blood culture-confirmed typhoid fever occurred in 12 children in the Vi-TCV group (46.9 per 100,000 person-years) and 62 children in the MenA group (243 per 100,000 person-years). Overall VE was 80.7% (95% confidence interval (CI): 64.2% to 89.6%) in an ITT analysis, and 83.7% (95% CI: 68.1%−91.6%) in a per-protocol analysis. In total, 130 serious adverse events occurred in the first 6 months after vaccination (52 in Vi-TCV group and 78 in MenA group), including 6 deaths (all in MenA group). No serious adverse event was considered by the investigator as related to study vaccination.

**Conclusions:**

Vi-TCV reduced blood culture-confirmed typhoid fever among Malawian children aged 9 months to 12 years. (Funded by the Bill & Melinda Gates Foundation; ClinicalTrials.gov number NCT03299426.)

Typhoid fever, a systemic febrile illness caused by *Salmonella enterica* serovar Typhi (*S. typhi*), is responsible for more than 9 million infections and over 110,000 deaths globally each year, with the highest disease burden among school-age and pre-school children.^1,2^ An estimated 1.2 million typhoid cases and 18,703 deaths occur annually in sub-Saharan Africa, with 383-843 cases per 100,000 person-years reported in some urban settings.^3–5^

The increased public health importance of typhoid fever across sub-Saharan Africa over the past decade is due in part to emergence and spread of several multidrug resistant (MDR; resistant to first-line agents chloramphenicol, ampicillin, and co-trimoxazole) *S. typhi* lineages, particularly H58 (clade 4.3.1) and H56 (clade 3.1.1).^6,7,8^ In Malawi and other countries in East and Southern Africa, MDR H58 *S. typhi* emerged in 2010 following its introduction from Asia,^9,7^ becoming the predominant blood-stream infection among adults and children, with 2% case fatality and 5% rate of small bowel perforation.^10,11,12^ Emerging antimicrobial resistance to fluoroquinolones has been documented in East Africa,^7,13^ Nigeria,^6^ and Democratic Republic of Congo.^14^ Extensively drug-resistant (XDR) typhoid, resistant to fluoroquinolones and 3^rd^ generation cephalosporins, is established in Pakistan.^12^ The dual threat in Africa of local emergence or introduction of untreatable XDR typhoid from Asia underscores the need for typhoid fever prevention.^15^

In 2018, the World Health Organization (WHO) recommended typhoid conjugate vaccine (TCV) for children 6 months through 15 years of age in countries with high incidence of disease or antimicrobial resistance.^16^ Typbar TCV^®^ (Bharat Biotech International) is a WHO-prequalified typhoid conjugate vaccine. The Typhoid Vaccine Acceleration Consortium (TyVAC) was launched in 2017 with the aim to accelerate Vi-TCV introduction in low-income settings. TyVAC is conducting large, randomized controlled efficacy trials of a Vi-TCV in diverse epidemiological settings in Malawi, Nepal, and Bangladesh.^17–19^ Here we present vaccine efficacy and safety results for Vi-TCV from the African continent,^20,21^ through 18–24 months of follow-up, from a clinical trial of single dose Vi-TCV in Blantyre, Malawi.^22^

## Methods

### Study design and participants

This is a single center, phase 3, double-blind, individually randomized active-controlled trial in two urban townships in Blantyre, Malawi. Detailed methods have been published.^22,23^ Briefly, a target of approximately 28,000 healthy children were enrolled, aged 9 months through 12 years residing within the urban townships of Ndirande and Zingwangwa, whose parents/guardians provided written consent, with no previous history of typhoid vaccination, and no acute illness or history of allergy or hypersensitivity. Written assent was required for children aged ≥8 years. HIV status was solicited verbally and confirmed, if positive, using the participant’s health-passport, where possible. Participants were recruited through government health centers and primary schools. Safety data (Adverse Events (AEs) and Serious Adverse Events (SAEs)) were prospectively recorded.

The study was approved by the Malawi National Health Sciences Research Committee (#17/07/1866); Malawi Pharmacy, Medicines, and Regulatory Authority (#3010201791); University of Maryland, Baltimore Institutional Review Board (#HP-00076625); University of Liverpool Research Ethics Committee (#2941).

### Randomization and masking

Participants were randomized at a 1:1 ratio to receive a single dose of Vi-TCV or control meningococcal group A conjugate vaccine (MenA), using block randomization with block sizes from 6–12. The random allocation sequence was generated by the blockrand package (version 1.3) in R (version 3.4.1) and concealed before randomization, which occurred in real time immediately before vaccination. Parents, guardians, participants, and study staff involved in screening, eligibility assessment, and follow-up were fully blinded to vaccine-group assignment. Unblinded nurses prepared and administered vaccine in a private area and had no further role in the study after vaccination.

### Procedures and vaccines

Typbar TCV^®^ consists of Vi polysaccharide conjugated to a tetanus toxoid protein carrier (25 μg per 0.5 ml dose). MenA (MenAfriVac, Serum Institute of India PVT Ltd) was given to children aged ≥1 year at a dose of 10 μg per 0.5 ml and 5 μg per 0.5 ml to children aged <1 year. Vaccines were administered intramuscularly in the left thigh (children aged <1 year) or left arm (children aged ≥ 1 year). Both Vi-TCV and MenA were co-administered with routine measles-rubella vaccine (in the right thigh) in children aged 9–11 months (Malawi Expanded Programme on Immunization). Bharat Biotech International Limited supplied the Typbar TCV vaccine free of charge.

### Enhanced fever and safety surveillance

All participants were monitored for 30 minutes after vaccination for immediate AEs. Enhanced passive surveillance for fever and SAEs was conducted at four primary health centers (Ndirande, Zingwangwa, Gateway, Nayo) in addition to Queen Elizabeth Central Hospital, a government referral hospital, where parents/guardians were instructed to bring unwell children at any time. Usual health service provision was also enhanced by telephone and community messaging to participants. Children presenting with febrile illness (subjective fever for ≥72 hours; axillary temperature ≥38°C; or hospitalization with history of fever of any duration), had blood-culture collected (5 mL <5-year old; 10 mL >=5 year old), and malaria Rapid Diagnostic Test (RDT). Antimicrobial resistance of *S. typhi* isolates was tested by disc-diffusion.^24^ Isolates showing pefloxacin-resistance had confirmatory ciprofloxacin e-test (BioMerieux), minimum inhibitory concentration >0.06mg/L indicating resistance. Hospital admission and antimicrobial treatment were at the facility clinician’s discretion. Participants with blood-culture -confirmed *S. typhi* were followed up biweekly, until asymptomatic, to monitor treatment response and outcome.

### Outcomes

The primary outcome was blood-culture -confirmed typhoid fever occurring at any time after vaccination. Vaccine efficacy (VE) was calculated as (1-IRR) × 100%, where IRR is the incidence rate ratio (ratio of incidence in the Vi-TCV group compared with the MenA group). Secondary outcomes were safety profiles of Vi-TCV and MenA measured by 1) the number of AEs detected in the first 30 minutes after vaccination, 2) the number of SAEs within 28 days after vaccination, and 3) the number of SAEs within 6 months after vaccination. For the primary evaluation of VE, all children were under enhanced passive surveillance for at least 18 months (21 February 2018 through 3 April 2020).

### Statistical analysis

Details of sample size and power calculations have been reported.^22^ Briefly, assuming 75% VE, the minimum number of cases needed to test the null hypothesis that the vaccine has no protective efficacy (i.e., VE≤0), with 90% power, was 30. The primary analysis to test VE was based on the intention-to-treat (ITT) principle, which included all randomized children who were vaccinated and all first episodes of blood-culture -confirmed typhoid fever occurring after vaccination. In the ITT analysis, the vaccine group was defined by the vaccine randomly assigned, not by the vaccine received. Per-protocol VE analysis included children who completed the study, without any protocol deviations, received the vaccine to which they were assigned, and accrued first episodes of blood-culture -confirmed typhoid fever at least 14 days after vaccination.

Due to the interruption of surveillance activities caused by COVID-19 (3 April 2020 onwards), a protocol amendment in August 2020 changed the primary analysis endpoint to 3 April 2020, encompassing 18–24 months follow-up per participant. The Data Safety Monitoring Board (DSMB) approved the amendment, as the study had reached the pre-specified number of typhoid cases and the prolonged disruption in surveillance would affect evaluations of incidence, cases prevented, and number needed to vaccinate. Full cohort blinded surveillance will continue until 30 Sept 2021 (minimum 36-month follow-up) for secondary longer-term efficacy and subgroup analyses.

The incidence rate was calculated as the number of first episodes of blood-culture -confirmed typhoid fever divided by the total follow-up time. Individual follow-up time was the smallest of the following: Time to first episode of typhoid fever; time to withdrawal, loss to follow-up, death, or relocation out of study area; or time to the end of the analysis period. The incidence rate ratio (IRR) was calculated as the ratio of the incidence rate in the Vi-TCV to the MenA group, and VE was calculated as (1-IRR) × 100%. Subgroup analysis was conducted to evaluate VE for sex, study site, and <5 years vs. ≥5 years of age at vaccination; Poisson regression with the interaction term between each pre-planned subgroup of interest and the vaccine group was used to compare VE across subgroups.

Absolute risk reduction was calculated as the risk of blood-culture -confirmed typhoid fever in the MenA group minus that in the Vi-TCV group. The number needed to vaccinate was calculated as 1/absolute risk reduction, representing the number of children who need to be vaccinated to prevent one additional blood-culture -confirmed case of typhoid fever. The cumulative incidence of typhoid fever for each vaccine group was presented using the Kaplan-Meier method; and VE was estimated at 12, 18, and 24 months after vaccination using the life table method. All analyses were performed according to the pre-specified statistical analysis plan, using Stata/SE (version 16). For full details of study design and conduct see the protocol at nejm.org.

## Results

### Trial participants

**Figure 1:**
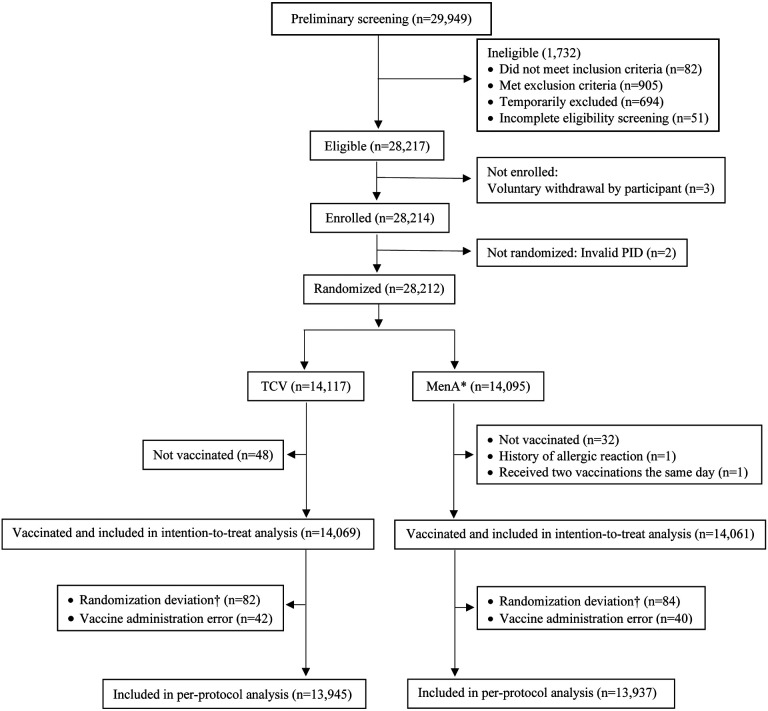
Disposition of participants (CONSORT flow diagram) *MenA group A meningococcal †Randomization deviation: Two participants received the same PID. The second participant who received a duplicate PID was assigned a new PID after randomization and excluded from per-protocol analysis.

From 21 February 2018 to 27 September 2018, 29,949 children underwent screening and 28,212 were randomly assigned to receive Vi-TCV or MenA vaccine (Figure 1). 28,130 children were vaccinated and included in the ITT analysis (14,069 in the Vi-TCV group and 14,061 in the MenA group) and 27,882 were included in the per-protocol analysis (13,945 in the Vi-TCV group and 13,937 in the MenA group) (Figure 1). Median age was 6 years (range: 0.8–12), and baseline characteristics were similar for the two study groups (Table 1).

### Vaccine efficacy

Between 21 February 2018 and 3 April 2020, 7,776 children presented to a passive surveillance center and met the primary case definition. Blood cultures were collected from 7,314 (94%). Seventy-five were positive for *S. typhi*. These included a 9-year-old with two typhoid fever episodes at 24 weeks and 49 weeks after vaccination; the second episode was therefore excluded from VE analyses. All 75 isolates (100%) were MDR, and 4/75 (5.3%) were ciprofloxacin-resistant.

**Figure 2.**
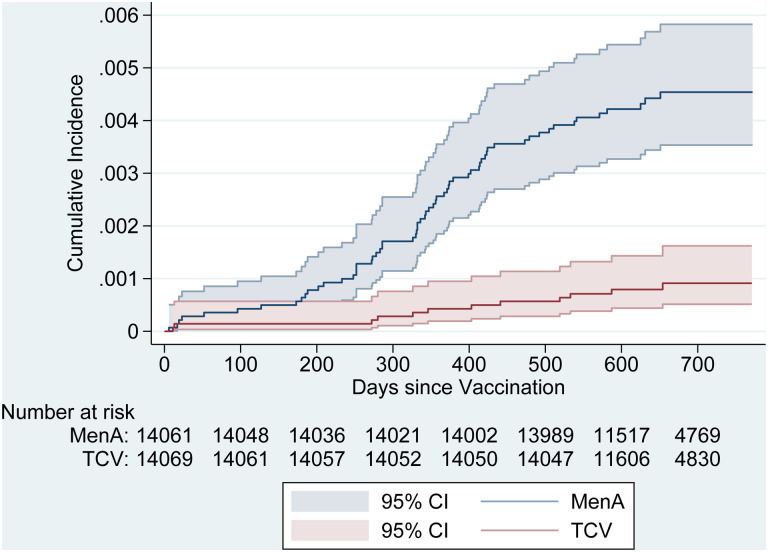
Kaplan-Meier Estimates of the Cumulative Incidence of Blood Culture-Positive Typhoid Fever, By Vaccine Group, Intention to Treat Population.

In the ITT analysis (Table 2), blood-culture -confirmed first episodes of typhoid fever (n=74) occurred in 12 children in the Vi-TCV group (incidence=46.9 cases per 100,000 person-years) and 62 children in the MenA group (incidence=243.2 cases per 100,000 person-years). One participant (MenA group) died from severe typhoid 7 months post-vaccination. VE for the primary outcome of blood-culture -confirmed typhoid fever, any time after vaccination, was 80.7% (95% CI: 64.2%−89.6%). The Kaplan-Meier curves show separation in cumulative incidence between Vi-TCV and MenA groups (p<0.001, Figure 2). The estimated VE was 84.6% (95% CI: 50%−94.4%), 82.9% (95% CI: 58.1%−92.5%), and 78.7% (95% CI: 52.8%−91.7%) at 12, 18, and 24 months after vaccination, respectively. The absolute risk reduction was 3.6 cases per 1,000 vaccinees, corresponding to a number needed to vaccinate of 278 to prevent one typhoid case. Three blood-culture -confirmed typhoid fever episodes occurring in the 14 days after vaccination were excluded from the per-protocol analysis, yielding an overall per-protocol VE of 83.7% (95% CI 68.1%−91.6%, Table 2).

For the ITT population, incidence of blood-culture -confirmed typhoid fever in the MenA group was similar across age groups (Table 2). Vi-TCV protective efficacy was 81.2% (95% CI: 54.8%−92.1%) for males and 80.3% (95% CI: 52.8%−91.8%) for females; and 77.9% (95% CI: 46.5%−90.9%) for Ndirande and 82.9% (95% CI: 59.2%−92.8%) for Zingwangwa. VE was similar between the <5 year and ≥5 year age groups (Table 2 and supplementary Figure 1).

### Safety

Three male participants in the MenA group had directly-observed AEs, all graded mild, within 30 minutes of vaccination. Two (skin rash and syncope) were deemed related to vaccination, and one (diarrhea) was deemed unrelated.

Within 28 days after vaccination, 14 SAEs occurred among 14 participants (12 females, 2 males); 4 in the Vi-TCV group (3 females) and 10 in the MenA group (9 females) (Table 3). Within 6 months of vaccination, 130 SAEs (52 in the Vi-TCV group and 78 in the MenA group) occurred in 118 participants (47 in the Vi-TCV group and 71 in the MenA group). Although more SAEs were observed among females than males in the MenA group (52/78 SAEs occurred in females), no sex difference was observed in the Vi-TCV group (28/52 SAEs occurred in females). The most common SAEs were respiratory tract infection, gastroenteritis, and malaria. One SAE (MenA group) was deemed by the investigator as possibly related to vaccination (fever, seizure, and neutrophilia one week after vaccination). Among 196 children identified as HIV-infected (89 in Vi-TCV group, 107 in MenA group), 190 were on antiretroviral therapy. Six deaths occurred within 6 months after vaccination (3 females); all were in the MenA group and deemed by the investigator as unrelated to vaccination.

## Discussion

In this field trial of Vi-TCV in Africa, a single dose Vi-TCV was efficacious in preventing typhoid fever among children 9 months to 12 years of age. The endemic typhoid incidence observed across both school-age and pre-school children in the control group (243/100,000 person-years) was reduced by 80.7% in the Vi-TCV group and Vi-TCV efficacy remained consistent across children <5 years or ≥5 years at vaccination, and throughout the observation period. Encouragingly, our VE (80.7% ITT; 83.7% per-protocol) in Malawi after 18–24 months is consistent with previously reported Vi-TCV VE of 81.6% after 12 months of follow-up among Nepalese children aged 9 months through 16 years of age.^17^ The safety profile was reassuring, with no excess SAEs in the Vi-TCV group and no AE or SAE considered related to Vi-TCV. The 6 deaths within the first 6 months occurred in the MenA group and the one death from typhoid, 7 months after vaccination, also occurred in the MenA group.

Typhoid vaccines have previously been trialed in Africa. Among 23,075 South African children aged 5 to 16 years, a single dose of unconjugated Vi capsular polysaccharide vaccine was 55–60% effective over 3 years in a randomized controlled trial.^25,26^ A systemic review and meta-analysis of randomized controlled trials, including trials and/or populations in Africa, showed cumulative efficacy at 3 years for the Ty21a oral and the polysaccharide Vi vaccine were similar at 51% (95% CI 36%, 62%) and 55% (95% CI 30%, 70%), respectively.^27^ Despite a 2008 WHO recommendation for programmatic use of existing typhoid vaccines in endemic countries,^28^ no African country integrated these vaccines into routine schedules, largely due to the unsuitability in the youngest children or the need for repeated doses. Typhoid burden in Malawi, and elsewhere in Africa, is high among both school and pre-school age children, down to one year of age.^29^ While these earlier typhoid vaccines were shown to be efficacious in school-age children, our study, importantly, demonstrates single dose Vi-TCV efficacy among African children <5 years of age, whose typhoid incidence was comparably high to that of school-age children. Vi-TCV efficacy was consistent throughout the study and ongoing typhoid surveillance of this cohort (until 36–42 months) will further assess the durability of protection and enable further age-stratified analyses in younger children. Analysis of a sub-study cohort is underway and will provide data on age-stratified immunogenicity.

Routine introduction of Vi-TCV among infants, coupled with catch-up campaigns to the age of 15 years, offers a strategy for typhoid control.^30^ WHO recommends vaccine introduction in countries with high incidence or emerging antimicrobial resistance. In February 2019, Zimbabwe^31^ deployed Vi-TCV programmatically in Africa as a local, targeted mass-vaccination campaign among children aged 6 months to 15 years in response to an antimicrobial-resistant typhoid outbreak. In Africa, routine introduction paired with catch-up campaigns are planned in Zimbabwe and Liberia.

Prevention of mother-to-child HIV transmission and successful national roll-out of antiretroviral treatment have dramatically reduced the prevalence of HIV among children in Malawi (1% among 0–4 year-olds and 1.5% among 0–14 year-olds in 2015–16).^32^ It is nonetheless reassuring that among 196 HIV-infected children identified and randomized in this study, no excess of SAEs was observed in the Vi-TCV group, and no SAEs were considered related to vaccination. In sub-Saharan Africa, HIV infection is, counterintuitively, epidemiologically associated with a 24-fold (95% CI: 9–100) reduction in diagnosis of blood culture-confirmed typhoid.^33,34^ The continued surveillance in this trial, along with an ongoing substudy assessing the immunogenicity of a one or two dose schedule of Vi-TCV among HIV-exposed children at 9- and 15-month immunization visits, will provide additional information in this vulnerable population.

MDR *S. typhi* remains prevalent in sub-Saharan Africa,^8^ typified by our finding of 100% resistance to first-line agents for suspected bloodstream infection. In the context of the rise in fluoroquinolone-resistance among *S. typhi* across Asia,^8^ the emergence in Malawi of four *S. typhi* strains with reduced susceptibility to fluroquinolones, from participants enrolled in this trial, is particularly concerning. Finally, the threat of independent emergence of azithromycin-resistant typhoid, as seen in several Asian countries,^35^ adds urgency and relevance to efforts to introduce this safe and efficacious Vi-TCV vaccine across the African continent and globally. Disclosure forms provided by the authors are available with the full text of this article at NEJM.org.

## Supplementary Material

Supplement

## Figures and Tables

**Table 1.** Baseline Characteristics of Children Enrolled in Efficacy Study of Typhoid Conjugate Vaccine (TCV), Intention to Treat Population.

Variable	TCV (N=14,069)	MenA* Vaccine (N=14,061)	Total (N=28,130)
Age at enrollment - years			
Mean (±SD)	6.1 ±3.3	6.2 ±3.3	6.1 ±3.3
Median (range)	6 (0.8–12)	6 (0.8–12)	6 (0.8–12)
Age group—no. (%)			
< 2years	1552 (11)	1598 (11.4)	3150 (11.2)
≥2 and <5years	3506 (24.9)	3581 (25.5)	7087 (25.2)
≥ 5years	9011 (64.1)	8882 (63.2)	17,893 (63.6)
Sex— no. (%)			
Female	7065 (50.2)	7231 (51.4)	14,296 (50.8)
Male	7004 (49.8)	6830 (48.6)	13,834 (49.2)
Study site— no. (%)			
Ndirande	8863 (63)	8832 (62.8)	17,695 (62.9)
Zingwangwa	5206 (37)	5229 (37.2)	10,435 (37.1)

* MenA - group A meningococcal.

**Table 2.** Occurrence of Blood Culture-Confirmed Typhoid Fever and Efficacy of Typhoid Conjugate Vaccine (TCV).

Variable	TCV			MenA Vaccine			Efficacy of TCV	Absolute Risk Reduction	Number Needed to Vaccinate††
	Participants (total follow-up time)	Cases	Incidence	Participants (total follow-up time)	Cases	Incidence			
	no. (person-years)		no. of cases/100,000 person-years (95% CI)	no. (person-years)		no. of cases/100,000 person-years (95% CI)	percent (95% CI)	per 1000 (95% CI)	no. (95% CI)
Intention-to-treat population (from time of vaccination)									
Age Group									
Total participants	14,069 (25,577)	12	46.9 (24.2–82)	14,061 (25,493)	62	243.2 (186.5–311.8)	80.7 † (64.2–89.6)	3.6 § (2.4–4.8)	277.8 (208.3–416.7)
<5years	5058 (9086)	5	55 (17.9–128.4)	5179 (9305)	20	215 (131.3–332)	74.4 (31.7–90.4)	2.9 (1–4.8)	344.8 (208.3–1000)
≥5years	9011 (16,491)	7	42.5 (17.1–87.5)	8882 (16,188)	42	259.5 (187–350.7)	83.7 (63.6–92.7)	4 (2.4–5.5)	250 (181.8–416.7)
Per-protocol population (14 days after vaccination)									
Age Group									
Total participants	13,945 (25,323)	10	39.5 (18.9–72.6)	13,937 (25,239)	61	241.7 (184.9–310.5)	83.7 † (68.1–91.6)	3.7 § (2.5–4.8)	270.3 (208.3–400)
<5years	5044 (9057)	5	55.2 (17.9–128.8)	5158 (9261)	20	216 (131.9–333.5)	74.4 (31.8–90.4)	2.9 (1.0–4.8)	344.8 (208.3–1000)
≥5years	8901 (16,267)	5	30.7 (10–71.7)	8779 (15,978)	41	256.6 (184.1–348.1)	88 (69.7–95.3)	4.1 (2.6–5.6)	243.9 (178.6–384.6)

* CI denotes confidence interval, and MenA group A meningococcal.

† p-value <0.001

§ p-value <0.001

Absolute risk reduction (=risk in MenA – risk in TCV) is the total reduction in risk of blood culture-confirmed typhoid fever that results from TCV vaccination.

†† The number of children needed to be vaccinated to prevent one case of blood-culture confirmed typhoid fever.

**Table 3.** Safety Outcomes by Vaccine Group, Intention to Treat Population.

Variable	TCV	MenA* Vaccine	Total
	**no. of participants**		
Total participants	14,069	14,061	28,130
Participants with serious adverse events within 28 days after vaccination	4	10	14
Participants with serious adverse events within 6 months after vaccination	47	71	118
	**no. of events observed**		
**Serious adverse events within 28 days after vaccination**	4	10	14
Infections and infestations	3	8	11
Other	1	2	3
**Serious adverse events within 6 months after vaccination**	52	78	130
Infections and infestations	34	55	89
Respiratory tract infection	16	21	37
Gastroenteritis	11	8	19
Malaria	4	10	14
Other infections	3	16	19
Nervous system disorders†	6	9	15
Injury, poisoning and procedural complications§	7	6	13
Other	5	8	13
**Deaths within 6 months after vaccination**	0	6 ǂ	6

* MenA group A meningococcal vaccine.

For the serious adverse events reported in this table, the assigned vaccine group is the same as the received vaccine group.

† Includes febrile convulsion and seizure.

§ Includes fractures and road traffic accidents.

ǂ 6 causes of death: Acute kidney injury secondary to hypovolemia; intra-abdominal infection secondary to shunt infection; death in the community with unknown cause; tracheoesophageal fistula after foreign body ingestion; massive trauma; chronic malnutrition complicated by possible TB, blood-culture negative sepsis or malignancy.
