# Supplementary material for: Safety and Efficacy of a Typhoid Conjugate Vaccine in Malawian Children
Source: N Engl J Med. Author manuscript. (PMC8202713; doi:10.1056/NEJMoa2035916)
Supplement: Supplement [file NEJMoa2035916_Supplement.pdf]

Supplementary Appendix

This appendix is provided by the authors to give readers additional information about their work.

Supplement to: Patel PD, Patel P, Liang Y, et al. Safety and Efficacy of a Typhoid Conjugate Vaccine in Children in Blantyre, Malawi: A Randomized, Double-Blinded Active-Controlled Phase 3 Trial. N Engl J Med.

Supplementary materials

Contents

1.1 Vaccines administered 2
1.2 Methods S1. Inclusion and exclusion criteria 2
1.2.1 Inclusion criteria[2] 2
1.2.2 Exclusion criteria[2] 2
1.3 Methods S2. Definitions 3
1.4 Supplementary Figure S1. Kaplan Meier showing the cumulative incidence of typhoid fever by age group 3
References 4

1.1 Vaccines administered

The trial vaccine, Vi polysaccharide-tetanus toxoid conjugate vaccine (Typbar TCV®, Bharat Biotech, Hyderabad, India) consists of Vi polysaccharide conjugated to a tetanus toxoid protein carrier[1] (25 µg per 0.5 ml dose). Vi-TCV was available as a 2.5 ml 5-dose vial with each 0.5 ml vaccine dose containing purified Vi-capsular polysaccharide of *S*. Typhi Ty2 conjugated to 25 mcg tetanus toxoid. Meningococcal capsular Group A conjugate vaccine (MenA, MenAfriVac, Serum Institute of India PVT Ltd) was given to children aged ≥1 year at a dose of 10 µg per 0.5 ml and 5 µg per 0. 5ml to children aged <1 year. Vaccines were administered intramuscularly in the left thigh (children aged <1 year) or left arm (children aged ≥ 1 year). Both Vi-TCV and MenA were co-administered with the routine measles-rubella vaccine (in the right thigh) in children aged 9-11 months (Malawi Expanded Programme on Immunization).

1.2 Methods S1. Inclusion and exclusion criteria

1.2.1 Inclusion criteria[2]

- Healthy male or female child at least 9 months of age and no older than 12 years and 364 days at the time of vaccination.
- Child whose parent or guardian resides within the study areas; Ndirande and Zingwangwa at the time of vaccination and who intends to be present in the area for the duration of the trial.
- A child whose parent or guardian has voluntarily given informed consent.

1.2.2 Exclusion criteria[2]

- History of documented hypersensitivity to any component of the vaccine.
- Prior receipt of any typhoid vaccine in the past 3 years.
- History of severe allergic reaction with generalized urticarial, angioedema, or anaphylaxis.
- Any condition determined by the investigator likely to interfere with evaluation of the vaccine or to be a significant potential health risk to the child or make it unlikely that the child would complete the study.

The following were temporary contraindications to enrollment and vaccination. If these applied, the participant was temporarily excluded from vaccination until 48 hours passed. A re-assessment was done to ensure these temporary exclusion criteria no longer existed:

- Reported fever within 24 hours before vaccination.
- Use of antipyretics within 4 hours before vaccination.

1.3 Methods S2. Definitions

An adverse event was defined as any untoward medical occurrence in a participant to whom Vi-TCV or MCV-A has been administered, including occurrences that are not necessarily caused by or related to the vaccine. The following adverse events were documented: fever, injection site pain, swelling, tenderness, erythema, myalgia, and arthralgia.

A serious adverse event was defined as any untoward medical occurrence that results in death; is life-threatening; requires inpatient hospitalization or prolongation of existing hospitalization; results in persistent or significant disability.

1.4 Supplementary Figure S1. Kaplan Meier showing the cumulative incidence of typhoid fever by age group

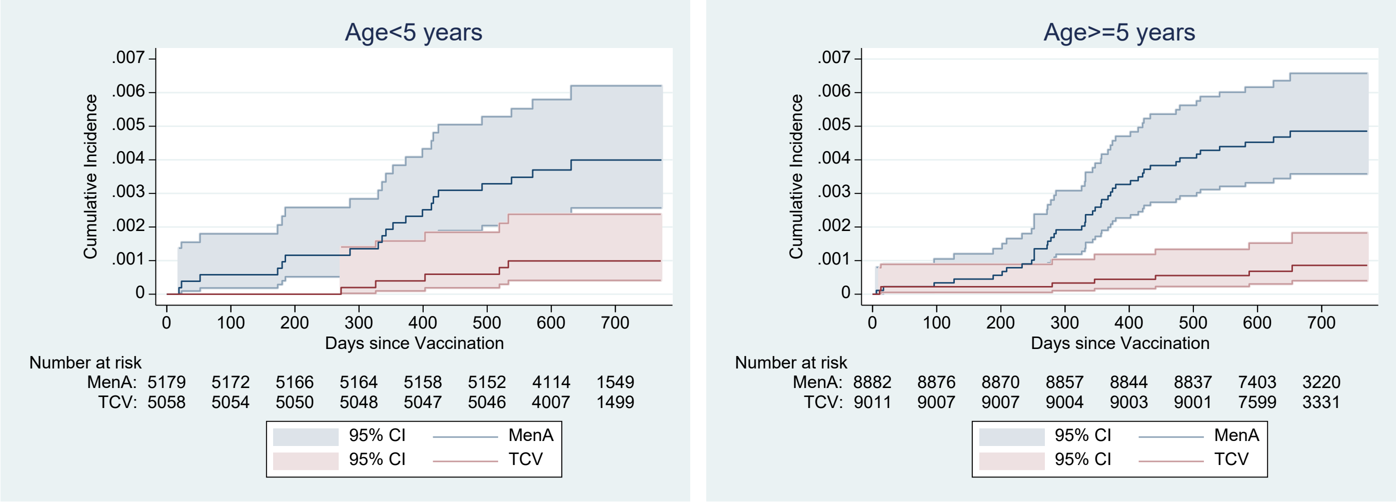

Supplementary appendix Figure S1. Kaplan-Meier Estimates the Cumulative Incidence of Blood Culture-Positive Typhoid fever by Vaccine Group and Age Group, Intention to Treat Population. Blood culture-positive typhoid fever was the primary outcome

References

1. WHO. Typhoid vaccines: WHO position paper. Wkly Epidemiol Rec 2008;83(6):49–59.

2. Meiring JE, Laurens MB, Patel P, et al. Typhoid vaccine acceleration consortium Malawi: A Phase III, randomized, double-blind, controlled trial of the clinical efficacy of typhoid conjugate vaccine among children in Blantyre, Malawi. Clin Infect Dis 2019;68(Suppl 2):S50–8.
